# Partial preservation of the normal thyroid gland based on tumor diameter may be possible in small medullary thyroid carcinoma: a two-center 15-year retrospective study

**DOI:** 10.3389/fonc.2023.1216394

**Published:** 2023-07-13

**Authors:** Guiming Fu, Xiaoyi Li, Fengli Guo, Xianhui Ruan, Wei Zhang, Weijing Zhang, Yaping Zhang, Yibo Chen, Chunhua Li, Jin Chen, Xiangqian Zheng, Zhaohui Wang, Ming Gao

**Affiliations:** ^1^ Department of Thyroid and Neck Tumor, Tianjin Medical University Cancer Institute and Hospital, National Clinical Research Center for Cancer, Key Laboratory of Cancer Prevention and Therapy, Tianjin’s Clinical Research Center for Cancer, Tianjin, China; ^2^ Thyroid-Otolaryngology Department, Sichuan Clinical Research Center for Cancer, Sichuan Cancer Hospital & Institute, Sichuan Cancer Center, Affiliated Cancer Hospital of University of Electronic Science and Technology of China, Chengdu, China; ^3^ Medical Diagnostic Radiology Department, Sichuan GEM Flower Hospital & North Sichuan Medical College, Chengdu, China; ^4^ Department of Thyroid and Breast Surgery, Binzhou Medical University Hospital, Binzhou, China; ^5^ School of Medicine, Nankai University, Tianjin, China; ^6^ School of Medicine, University of Electronic Science and Technology of China, Chengdu, China; ^7^ Department of Thyroid and Breast Surgery, Tianjin Union Medical Center, Tianjin, China; ^8^ Tianjin Key Laboratory of General Surgery in Construction, Tianjin Union Medical Center, Tianjin, China

**Keywords:** thyroid malignancy, medullary thyroid carcinoma, tumor diameter, surgery, prognosis

## Abstract

**Background:**

At present, there are some controversies in the formulation of surgical protocol for small medullary thyroid carcinoma(s-MTC). We wanted to explore the feasibility of normal thyroid gland retention in small medullary thyroid carcinoma based on different tumor diameters and its prognostic impact on the tumor.

**Methods:**

The data of patients with stage T1 MTC treated at Tianjin Cancer Hospital and Sichuan Cancer Hospital from 2006 to 2021 were analyzed. The tumor diameters of 0.5 cm and 1.0 cm were used as dividing points. The outcomes were tumor recurrence, metastasis, or patient death. Survival was estimated by the Kapan–Meier curve.

**Results:**

A total of 121 T1 s-MTC patients were included, including 55 with total thyroidectomy (TT) and 66 with subthyroidectomy (Sub-TT). There were eleven cases of tumor recurrence and metastasis, and four patients died. When the tumor diameter was 1.0 cm as the cut-off point, tumor diameter (p = 0.010), TT (p = 0.028), unilateral and bilateral type (p = 0.009), and TNM staging (p = 0.007) had significant effects on progression-free survival (PFS). The tumor diameter, unilateral and bilateral type, and TT were risk factors for the prognosis of T1 MTC (p < 0.05).

**Conclusion:**

The tumor diameter of 1.0 cm can be used as a cut-off point for stage T1 MTC. Alt-hough there was no significant difference in overall survival (OS) between T1a and T1b in patients, tumor diameter significantly influenced PFS. TT is not necessary for patients with sporadic MTC with T1a.

## 1 Introduction

MTC was first proposed by Hazand et al. in 1959 as a single pathological type of thyroid neuroendocrine carcinoma. It originates from parafollicular thyroid C cells and can be divided into sporadic and hereditary types according to their heredity. Although the incidence of MTC is less than 5% of all thyroid cancers, it accounts for 13.0% of all thyroid cancer-related deaths (1–3) and is mainly treated by surgical removal of all tumor tissue (4). In 2005, the National Comprehensive Cancer Network (NCCN) published an article that advocated at least TT and bilateral central dissection for all patients with MTC (5). Revised guidelines from the American Thyroid Association (ATA) in 2015 recommended TT and central lymph node dissection for patients with MTC in whom cervical lymph node metastasis or distant metastasis was not found during ultrasound (3, 4). In 2019, the European Society for Medical Oncology (ESMO) recommended that surgical regimens be based on calcitonin levels in patients with MTC. It is considered that only partial normal thyroid tissue can be retained for unilateral lobular MTC with negative cervical lymph nodes, indicated by preoperative color ultrasound, undetectable serum calcitonin after surgery, and no strain RET mutation (6). As of 2022, the NCCN thyroid cancer guidelines still recommend at least TT and bilateral central resection for all patients with tumors ≥1.0 cm in diameter or with cancer foci in bilateral thyroid lobes. TT and central cervical dissection are also recommended for patients with only unilateral thyroid lesions and tumor diameter <1.0 cm (7). Thus, the world’s leading guidelines for the treatment of MTC are cautious about whether to retain part of the thyroid gland, preferring to perform at least TT and bilateral central cervical lymph node dissection, regardless of the stage of the primary tumor. In the past decade or more, we have found that an increasing number of patients with MTC are accidentally discovered during physical examination. According to our experience, these patients tend to have small tumor lesions, and most of them are not willing to accept lifelong medication after TT, but the overall prognosis of the disease is good. At the same time, the concept of thyroid microcarcinoma is widely accepted and some reports indicate that TT may not be necessary for early MTC with a small tumor diameter (8, 9). Therefore, in search of relevant information, we retrospectively analyzed and summarized the data of patients with stage T1 MTC treated at two major cancer centers in southern China and northern China over the past 15 years.

## 2 Materials and methods

### 2.1 Patients

Clinical and pathological data of patients with stage T1 MTC treated at Tianjin Cancer Hospital and Sichuan Cancer Hospital from November 2006 to November 2021 were retrospectively analyzed, and data of eligible cases were collected, sorted, and statistically analyzed according to inclusion and exclusion criteria. Inclusion criteria: (1) postoperative pathological findings confirmed MTC; (2) all patients were newly diagnosed and initially treated, and had no previous history of thyroid surgery; (3) color Doppler ultrasound indicated that the maximum diameter of cancer nodules was less than 2.0 cm, with or without cervical lymph node metastasis; (4) no extraocular invasion of cancer nodules was found by preoperative color Doppler ultrasound and intraoperatively; (5) all surgeons had more than 10 years of experience in thyroid surgery; (6) the scope of surgery at least included the removal of the affected glandular lobe and isthmus, with or without the dissection of the neck lymph node; (7) complete clinical and pathological data of patients and long-term follow-up. Exclusion criteria: (1) postoperative pathology indicated papillary carcinoma, follicular carcinoma, or mixed carcinoma of other pathological types; (2) other malignant tumors in the past; (3) refusal to be studied; (4) lost to follow-up.

### 2.2 Range of cervical lymph node dissection

Lymph nodes in the central cervical region were dissected (zones VI and VII): upper boundary—lower margin of the hyoid; lower boundary—superior sternal fossa; outer boundary—lateral margins of the carotid sheath; bottom boundary—prevertebral fascia. Lymph nodes in the lateral cervical region were dissected in areas II–V: upper boundary—mastoid plane; lower boundary—supraclavicular fossa; inner boundary—sternocleidomastoid medial border; outer boundary—trapephalus anterior border; bottom boundary—prevertebral fascia.

### 2.3 Postoperative follow-up

All patients were followed up at the outpatient clinic after surgery, and the examination items mainly included blood tests (thyroid function, serum calcitonin, carcinoembryonic antigen) and imaging examinations (neck color Doppler ultrasound, chest thin layer computed tomography). Blood tests and color Doppler ultrasonography of the neck were performed every three months for the first year after surgery and then every six months. Thin-section computed tomography of the chest was performed semiannually or annually as needed. Total thyroidectomy and central or lateral cervical lymph node dissection were performed for patients with significantly elevated serum calcitonin in a short period of time or with definite recurrent lesions confirmed by color Doppler ultrasound. Multidisciplinary treatment should be made for patients with distant metastasis such as lung metastasis.

### 2.4 Grouping and statistics

We used the tumor diameters of 0.5 cm and 1.0 cm as the cut-off points for two groups. OS and PFS were the main outcome events. Classification variables using frequency and percentage were described, using the χ2 test or Fisher’s exact test for comparison. Continuous variables were described using mean ± standard deviation. The Shapiro–Wilk test was used to verify normal distribution, and the independent sample t-test was used for comparison between groups. Survival was estimated by the Kaplan–Meier curve and compared with the log-rank test. Univariate analysis of different disease characteristics was carried out with the Cox hazards model. Disease characteristics with p ≤ 0.20 were included in multivariate prognostic hazard ratio analysis to determine independent risk factors. Bilateral p < 0.05 indicated statistical significance. All analyses were performed with SPSS software version 25.0 (SSPS, Inc., Chicago, Illinois, US).

## 3 Results

A total of 121 patients in the T1 stage met the inclusion criteria, including 22 patients with the familial type (18.18%) and 99 patients with the sporadic type (81.82%). There were 44 males (36.36%) and 77 females (63.64%), with a male-to-female ratio of 1:1.75. The age range was 24 to 78 years old, with an average age of 49.79 ± 11.38 years old. The maximum diameter of cancer foci was 0.30–1.90 cm, with an average of 1.18 ± 0.51 cm. There were 92 cases (76.03%) of single-lobe carcinoma and 29 cases (23.97%) of multi-lobe carcinoma, of which 20 cases were single-lobe multifocal carcinoma and 9 cases were double-lobe multifocal carcinoma. TT was performed in 55 cases (45.45%) and Sub-TT in 66 cases (54.55%). Central cervical lymph nodes were dissected in 103 cases (85.12%), including 60 unilateral cases and 43 bilateral cases. There were 53 cases (43.80%) with lateral cervical lymph node dissection, 47 with unilateral lymph node dissection, and 6 with bilateral lymph node dissection. Twenty patients (16.53%) with central cervical lymph node metastasis and forty-three patients (35.54%) with lateral cervical lymph node metastasis were diagnosed postoperatively. Tumor staging was performed according to the International Union for Cancer Control (UICC) Version 8 staging system, including stage I (39 cases, 32.23%), stage III (19 cases, 15.70%), stage IV (44 cases, 36.36%), and uncertain stage (19 cases, 15.70%) (**Table 1**). The follow-up time was 3 to 178 months. Eleven patients (9.10%) developed tumor recurrence and metastasis, and four patients (3.31%) died. The 5-year, 10-year, and 15-year OS rates of stage T1a patients were all 100.00%, and the PFS rates were 98.11%, 96.23%, and 96.23%, respectively. The 5-year, 10-year, and 15-year OS rates of stage T1b patients were 95.59%, 95.59%, and 94.12%, respectively. The PFS rates of stage T1b patients were 88.24%, 85.29%, and 85.29%, respectively.

**Table 1:** Univariate analysis of groups was performed using 1.0 cm as the tumor diameter cut-off point.

Variables		Patients (No. (%))	Tumor Diameter (cm)		χ^2^/t	*p*-Value
			≤1.0	>1.0, <2.0		
Number of patients		121 (100.00)	53 (43.80)	68 (56.20)		
Age (Mean ± SD)		49.79 ± 11.38	49.70 ± 10.83	49.85 ± 11.86	0.074	0.941
Sex					2.649	0.104
	Male	44 (36.36)	15 (28.30)	29 (42.65)		
	Female	77 (63.64)	38 (71.70)	39 (57.35)		
Type					4.297	0.038
	Hereditary	22 (18.18)	14 (26.42)	8 (11.76)		
	Sporadic	99 (81.82)	39 (73.58)	60 (88.24)		
Unilateral or bilateral					–	0.005
	Unilateral	112 (92.56)	53 (100.00)	59(86.76)		
	Bilateral	9 (7.44)	0	9(13.24)		
Multifocal					8.277	0.004
	Yes	28 (23.14)	6 (11.32)	22 (32.35)		
	No	93 (76.86)	47 (88.68)	46 (67.65)		
Edge of nodules					2.605	0.107
	Regular	24 (19.83)	7 (13.21)	17 (25.00)		
	Irregular	97 (80.17)	46 (86.79)	51 (75.00)		
Boundary of nodules					2.349	0.125
	Clear	41 (33.88)	14 (26.42)	27 (39.71)		
	Unclear	80 (66.12)	39 (73.58)	41 (60.29)		
Nodules blood supply					24.717	<0.001
	Rich	56 (46.28)	11 (20.75)	45 (66.18)		
	Not rich	65 (53.72)	42 (79.25)	23 (33.82)		
Calcification					0.646	0.422
	Yes	94 (77.69)	43 (81.13)	51 (75.00)		
	No	27 (22.31)	10 (18.87)	17 (25.00)		
Lymphatic metastasis					10.453	0.015
	Nx	19 (15.70)	12 (22.64)	7 (10.29)		
	N0	39 (32.23)	22 (41.51)	17 (25.00)		
	N1a	20 (16.53)	7 (13.21)	13 (19.12)		
	N1b	43 (35.54)	12 (22.64)	31 (45.59)		
Distant metastasis					–	0.504
	Yes	2 (1.65)	0	2 (2.94)		
	No	119 (98.35)	53 (100.00)	66 (97.06)		
TT					6.809	0.009
	Yes	55 (45.45)	17 (32.08)	38 (55.88)		
	No	66 (54.55)	36 (67.92)	30 (44.12)		
8th AJCC/TNM stage					10.69	0.014
	Uncertain	19 (15.70)	12 (22.64)	7 (10.29)		
	I	39 (32.23)	22 (41.51)	17 (25.00)		
	III	19 (15.70)	7 (13.21)	12 (17.65)		
	IV	44 (36.36)	12 (22.64)	32 (47.06)		
PFS					–	0.022
	Yes	110 (90.91)	52 (98.11)	58 (85.29)		
	No	11 (9.10)	1 (1.89)	10 (14.71)		
OS					–	0.13
	Alive	117 (96.69)	53 (100.00)	64 (94.12)		
	Dead	4 (3.31)	0	4 (5.88)		
Survival months (Mean ± SD)		66.30 ± 33.05	71.34 ± 28.87	62.37 ± 35.69	1.489	0.139

SD, standard deviation; TT, total thyroidectomy; AJCC, American Joint Committee on Cancer; TNM, tumor node metastasis; PFS, progression-free survival; OS, overall survival.

**Figure 1:**
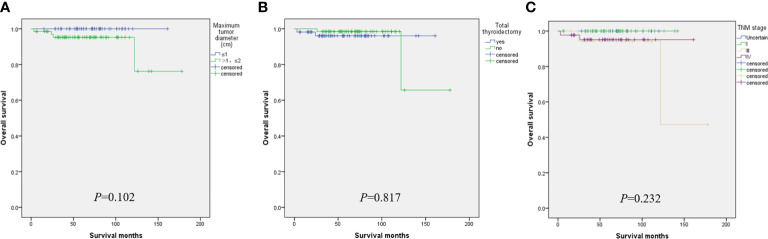
When the maximum tumor diameter was 1.0 cm as the cut-off point, Kaplan–Meier curve survival analysis showed that maximum tumor diameter **(A)**, total thyroidectomy **(B)**, and TNM staging **(C)** had no significant effect on OS.

**Figure 2:**
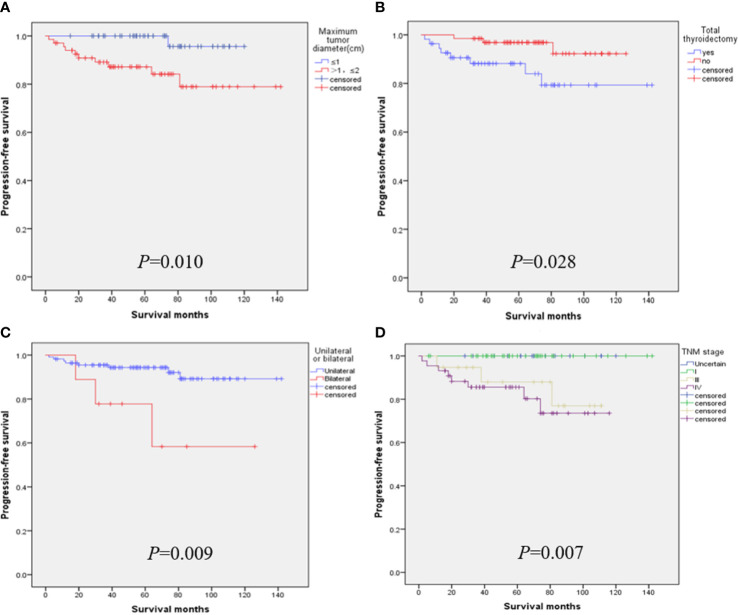
When the maximum tumor diameter was 1.0 cm as the cut-off point, Kaplan–Meier curve survival analysis showed that maximum tumor diameter **(A)**, total thyroidectomy **(B)**, unilateral and bilateral type **(C)**, and TNM staging **(D)** all had significant effects on PFS.

When the tumor diameter of 1.0 cm was taken as the cut-off point, χ2 inspections or t-tests showed that for cancer patients with T1a and T1b, the onset type (p = 0.038), single and double side (p = 0.005), multifocal carcinoma (p = 0.004), focal blood supply (p < 0.001), lymph node metastasis (p = 0.015), the total removal of the thyroid (p = 0.009), TNM staging (p = 0. 014), and PFS (p = 0.022) were statistically significant for OS (**Table 1**). Kaplan–Meier curve estimated survival rates showed that tumor diameter (p = 0.102), TT (p = 0.817), and TNM staging (p = 0.232) had no significant impact on OS in patients with MTC at T1 (**Figure 1**). At the same time, however, we found that tumor diameter (p = 0.010), TT (p = 0.028), unilateral and bilateral type (p = 0.009), and TNM staging (p = 0.007) all had significant effects on PFS (**Figure 2**). Univariate analysis showed that tumor diameter (p = 0.034), unilateral and bilateral type (p = 0.018), and TT (p = 0.042) were prognostic risk factors for T1 MTC. All factors with p ≤ 0.20 were included in the Cox hazards model for multi-factor analysis, but no independent risk factors were found (p > 0.05) (**Table 2**). With a tumor diameter of 0.5 cm as the boundary point, χ2 inspections or t-tests showed that between the two groups of patients, there was no significant difference (p > 0.05) in the onset type, single and double side, multifocal carcinoma, calcification, or lymph node metastasis (**Table 3**). Kaplan–Meier curves did not identify disease characteristics that were significant for OS and PFS. No positive or independent risk factors were found in univariate analysis and multivariate analysis using the Cox hazards model.

**Table 2:** Univariate analysis of groups was performed using 0.5 cm as the tumor diameter cut-off point.

Variables		Patients [No. (%)]	Tumor Diameter (cm)		χ^2^/t	*p*-Value
			≤0.5	>0.5, <1.0		
Number of patients		53 (100.00)	17 (32.08)	36 (67.92)		
Age (Mean ± SD)		49.70 ± 10.83	48.41 ± 10.23	50.31 ± 11.20	0.59	0.558
Sex					–	1
	Male	15 (28.30)	5 (29.41)	10 (27.78)		
	Female	38 (71.70)	12 (70.59)	26 (72.22)		
Type					–	0.336
	Hereditary	14 (26.42)	6 (35.29)	8 (22.22)		
	Sporadic	39 (73.58)	11 (64.71)	28 (77.78)		
Unilateral or bilateral					–	–
	Unilateral	53 (100.00)	17 (100.00)	36 (100.00)		
	Bilateral	0	0	0		
Multifocal					–	1
	Yes	6 (11.32)	2 (11.76)	4 (11.11)		
	No	47 (88.68)	15 (88.24)	32 (88.89)		
Edge of nodules					–	0.408
	Regular	7 (13.21)	1 (5.88)	6 (16.67)		
	Irregular	46 (86.79)	16 (94.12)	30 (83.33)		
Boundary of nodules					–	0.506
	Clear	14 (26.42)	3 (17.65)	11 (30.56)		
	Unclear	39 (73.58)	14 (82.35)	25 (69.44)		
Nodules blood supply					–	0.469
	Rich	11 (20.75)	2 (11.76)	9 (25.00)		
	Not rich	42 (79.25)	15 (88.24)	27 (75.00)		
Calcification					–	0.709
	Yes	43 (81.13)	13 (76.47)	30 (83.33)		
	No	10 (18.87)	4 (23.53)	6 (16.67)		
Lymphatic metastasis					3.015	0.388
	Nx	12 (22.64)	6 (35.29)	6 (16.67)		
	N0	22 (41.51)	7 (41.18)	15 (41.67)		
	N1a	7 (13.21)	2 (11.76)	5 (13.89)		
	N1b	12 (22.64)	2 (11.76)	10 (27.78)		
Distant metastasis					–	–
	Yes	0	0	0		
	No	53(100.00)	17 (100.00)	36 (100.00)		
TT					0.082	0.775
	Yes	17(32.08)	5 (29.41)	12 (33.33)		
	No	36(67.92)	12 (70.59)	24 (66.67)		
8th AJCC/TNM stage					3.015	0.388
	Uncertain	12 (22.64)	6 (35.29)	6 (16.67)		
	I	22 (41.51)	7 (41.18)	15 (41.67)		
	III	7 (13.21)	2 (11.76)	5 (13.89)		
	IV	12 (22.64)	2 (11.76)	10 (27.78)		
PFS					–	1
	Yes	52 (98.11)	17 (100.00)	35 (97.22)		
	No	1 (1.89)	0	1 (2.78)		
OS					–	–
	Alive	53 (100.00)	17 (100.00)	36 (100.00)		
	Dead	0	0	0		
Survival months (Mean ± SD)		71.34 ± 28.87	80.06 ± 26.11	67.22 ± 29.53	1.531	0.132

SD, standard deviation; TT, total thyroidectomy; AJCC, American Joint Committee on Cancer; TNM, tumor node metastasis; PFS, progression-free survival; OS, overall survival.

**Table 3:** Multivariate prognostic hazard ratio analysis was performed using the Cox proportional hazards model.

	Univariate Analysis			Multivariate Analysis		
	Hazard Ratio	95% CI	*p*-Value	Hazard Ratio	95% CI	*p*-Value
Tumor diameter (cm)	0.108	0.014–0.845	0.034	0.168	0.019–1.512	0.111
Unilateral or bilateral	0.201	0.053–0.759	0.018	1.614	0.252–10.339	0.613
Multifocal	0.329	0.100–1.084	0.068	1.256	0.248–6.376	0.783
Edge of nodules	1.63	0.432–6.151	0.471	–	–	–
Nodules blood supply	0.301	0.080–1.138	0.077	1.236	0.284–5.386	0.777
Boundary of nodules	1.459	0.444–4.792	0.533	–	–	–
Calcification	0.757	0.163–3.509	0.722	–	–	–
TT	3.979	1.052–15.046	0.042	0.378	0.091–1.574	0.378
N stage	**-**	**-**	0.942	–	–	–
M stage	20.676	0.000–1.091E + 10	0.768	–	–	–
8th AJCC/TNM stage	**-**	**-**	0.959	–	–	–

TT, total thyroidectomy; N, node; M, metastasis; AJCC, American Joint Committee on Cancer; TNM, tumor node metastasis.

## 4 Discussion

### 4.1 Incidence of s-MTC patients

Since 1988, when the World Health Organization defined thyroid cancer with the largest tumor diameter ≤1.0 cm as microcarcinoma, extensive research on thyroid micro-carcinoma has been carried out worldwide. As early as 1998, Beressi N. et al. reported that the proportion of sporadic micro MTC in all sporadic MTC increased rapidly, among which the proportion was 3.6% before 1984, 14.3% in 1984–1989, and 22.0% in 1990–1996 (10). Kazaure H.S. et al. analyzed 310 cases of medullary thyroid micro-carcinomas detected between 1988 and 2007 and found that the proportion of micro MTC increased by 39.0% (11). Similarly, Machens et al. found that the incidence of micro MTC increased from 19.0% to 39.0% between 2011 and 2015, and Kwon H et al. reported similar findings (12, 13). Our study showed that T1 MTC accounted for 1.33% of the total MTC from 2006 to 2010, 12.26% from 2011 to 2015, and 26.25% from 2016 to 2021, and morbidity also showed the same trend. It can be seen that the incidence of patients with small-sized MTC continues to rise.

### 4.2 Influence of tumor diameter on the prognosis of patients with s-MTC

Overall, the incidence of MTC in thyroid cancer is consistently below 5%, with a 5-year relative survival rate of about 93.0% for stage I–III patients and 28% for stage IV patients (14–17). Kim S.J. reported a 95.0% OS rate after 71 months of follow-up in patients with minimal MTC (18). Beressi N. reported a 10-year survival rate of 93.9% ± 4.4% for micro MTC (10). Kazaure H.S. et al. reported that overall 10-year survival rates were 96.0%, 87%, and 50% for patients with focal lesions, regional metastases, and distant metastases in minimal MTC, respectively (11). However, Saltiki K.’s study showed that in patients with tumor sizes of 0.1–1.0 cm and 1.1–1.5 cm, the probability of no disease progression at 10 years was 96.6% and 81.3%, respectively, and suggested that tumor size may only be clinically significant in patients with MTC of 1.0 cm (19). In this study, survival analysis of patients with stage T1 MTC was conducted with tumor diameters of 0.5 cm and 1.0 cm as the cut-off points. The results showed that the 5-year, 10-year, and 15-year OS rates of T1a patients with T1 MTC were all 100.00%, and PFS rates were 98.11%, 96.23%, and 96.23%, respectively. Meanwhile, the 5-year, 10-year, and 15-year OS rates of stage T1b patients were 95.59%, 95.59%, and 94.12%. The PFS rates of stage T1b patients were 88.24%, 85.29%, and 85.29%.

Thus, it is highly effective to use a 1.0 cm tumor diameter as the cut-off point. Although it is not significant for the OS of patients, it is critical for the PFS of the disease. It can be seen that the PFS of patients with T1a is significantly higher than that of patients with T1b (p = 0.010). When the tumor diameter of 0.5 cm was used as the cut-off point, we did not obtain valuable information on disease survival, but we believe that this may be related to the small number of patients with a tumor diameter of less than 0.5 cm. Generally, the prognosis of MTC at this stage is still good, and patients with s-MTC still have high OS and PFS rates.

### 4.3 Controversy over TT in patients with s-MTC

Although the idea of TT and bilateral central cervical lymph node dissection for MTC patients has long been accepted by most surgeons, an increasing number of patients with early MTC are hoping to retain a normal thyroid gland. We may have overstated the benefits of TT for patients with early MTC and overlooked the potential risks of surgical complications and lifelong levothyroxine tablets, especially in poor populations. Raffel A. et al. performed a second surgery on an s-MTC patient who was found by chance in postoperative pathology and who was more likely to develop temporary and permanent hypoparathyroidism after surgery (5.6%; 3.5–8.8%), vocal cord paralysis (3.8–8.5%; 2.8–8.4%), and transient Horner syndrome (3.8–5.6%) (20). Follow-up results showed a 100% biochemical cure rate within 1.5–10.0 years after Sub-TT. Therefore, it is suggested that TT and neck dissection should not be mandatory for patients with sporadic and isolated T1 MTC as long as the genetic background is excluded (20). Three different reports by Miyauchi A., Randle R.W., and Shabina R. analyzed a total of 927 cases of sporadic s-MTC, and all concluded that unilateral thyroidectomy was acceptable for sporadic isolated s-MTC patients with normal contra-lateral thyroid and no RET mutation detected in the reproductive cell line. Dis-ease-specific survival rates did not change (21–23).

Our team has also reached similar conclusions in previous studies on surgical selection and prognosis of unilateral sporadic medullary thyroid carcinoma, i.e., that patients with total thyroidectomy have little benefit in terms of biochemical cure/OS (24). Hamy A. and Zhang D. even argued that sporadic minimal MTC was almost 100% located in the thyroid gland, lymph node metastasis was rare, calcitonin could hardly be detected after surgery, and the value of central cervical dissection was questionable (16, 25). After a follow-up of 233 patients with MTC for 7 to 445 months, Ito et al. also considered it controversial to perform TT and lymph node dissection for all MTCS with lesions confined to the thyroid, especially for T1a (26). Interestingly, studies from the Korean NHIS database showed that a total of 34.6% of patients with MTC underwent adeno lobectomy alone, and this proportion did not change significantly between 2004 and 2016 (17). Similarly, our results also showed that TT had no significant effect on OS in T1a and T1b MTC patients, but it did significantly affect PFS (p = 0.028). Patients who underwent TT had a lower PFS rate (**Figure 1B**), which we believe is related to cervical lymph node metastasis. TT was performed for the included T1 patients mainly because most of them had already developed cervical lymph node metastasis or even distant metastasis when the disease was discovered. Therefore, even after TT, they still had a lower PFS rate, which was consistent with the lower PFS rate for the later TNM stage (**Figure 2D**). In addition, after TT, patients need to take levothyroxine tablets for lifelong replacement therapy, and the long-term potential risk of cardiovascular dis-ease and the economic burden should be considered before surgery.

### 4.4 Other influencing factors of MTC prognosis

Compared with differentiated thyroid carcinoma, MTC appears to be more prone to cervical lymph node metastasis and distant metastasis and may be associated with many other factors in addition to tumor diameters, such as male sex, calcitonin, and carcinoembryonic antigen levels, RET mutations, bilateral carcinoma, multifocal carcinoma, neoplasmic thyroid extracapsular invasion, marginal burrs, morphological irregularities, calcification, and blood supply (26–31). The level of calcitonin before and after surgery and the doubling time of calcitonin after surgery is now widely believed to be associated with the prognosis of MTC and determines whether the surgeon decides to perform elective lateral cervical lymph node dissection at the time of initial surgery (32). For disease progression, postoperative calcitonin has been shown to be a more important predictor than tumor size (19). Due to the limitations of hospital testing levels, the cancer centers involved in this study did not carry out the detection of calcitonin levels 15 years ago. Meanwhile, since most of the small medullary thyroid carcinomas were accidentally found during the pathological examination after surgery, the level of calcitonin was not routinely detected before surgery.

**Figure 3:**
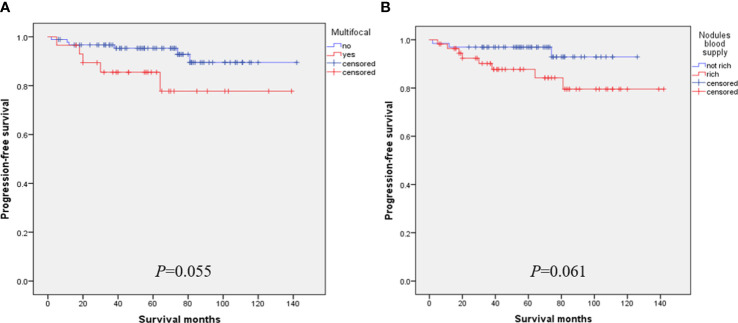
The effect of multifocal **(A)** and nodular blood supply **(B)** on PFS of s-MTC.

For advanced MTC patients with unresectable tumor recurrence or distant metastasis, patients with RET mutation or fusion will likely receive RET inhibitor therapy and other measures (33, 34). Bilateral carcinoma also appears to be a factor in poor prognosis. Bilateral cancers tend to be more prone to cervical lymph node and blood route metastasis than unilateral cancers. The results of this study were further confirmed in stage T1 MTC, where bilateral carcinoma had significantly lower PFS (**Figure 2C**). Multifocal carcinoma and nodular blood supply may be other factors affecting the PFS rate of s-MTC. Although the results of our study were negative, this may be due to the small number of patients analyzed (**Figure 3**). Cervical lymph node metastasis is another prognostic factor, and the later the stage of lymph node metastasis, the higher the recurrence rate. In particular, stage N1b patients had a significantly higher recurrence rate than those without cervical lymph node metastasis (13). Finally, the adhesion hyperplasia found in the tumor during the histopathological examination may also indicate that MTC has high invasive and metastatic potential. If adhesion hyperplasia is absent or only slightly visible, the risk of metastasis may be considered low (35, 36).

## 5 Conclusion

The incidence of s-MTC in patients with MTC continues to increase, and high cure rates can be achieved by early surgical excision. The tumor diameter of 1.0 cm could be used as the cut-off point for T1 MTC. Although there was no significant difference in OS rates between T1a and T1b patients, the former had a higher PFS rate. TT is not necessary for patients with sporadic s-MTC, but the preservation of part of the normal gland is careful, especially when the tumor diameter is greater than 1.0 cm, and the patient must be evaluated comprehensively before surgery.

## Data availability statement

The original contributions presented in the study are included in the article/supplementary material. Further inquiries can be directed to the corresponding authors.

## Author contributions

Conceptualization, GF and FG; methodology, GF and XL; software, XL; formal analysis, GF; investigation, GF, FG, WZ, WJZ, YZ, and YC; resources, ZW and XZ; data curation, XL; writing—original draft preparation, GF, XL, and FG; writing—review and editing, XR; visualization, XR; supervision, CL, JC, and XZ; project administration, ZW and XZ; funding acquisition, MG and XZ. All authors contributed to the article and approved the submitted version.
